# When washing is not enough: a cross-disciplinary analysis of hygiene and handling practices among vegetable traders in Nigeria

**DOI:** 10.1007/s12571-025-01531-x

**Published:** 2025-03-29

**Authors:** Itohan Ebunoluwa Abatan, Lenis Saweda O. Liverpool-Tasie, Adewale Olusegun Obadina

**Affiliations:** 1 Department of Food Science and Technology, Federal University of Agriculture, Abeokuta, Nigeria; 2 Department of Agricultural, Food, and Resource Economics, Michigan State University, East Lansing, MI USA; 3 Department of Biotechnology and Food Technology, University of Johannesburg, Johannesburg, South Africa

**Keywords:** Vegetable, Traders, Handling practices, Food safety, Microbial contaminants

## Abstract

Though improper food trader hygiene and handling practices can cause food contamination, few studies have examined both the drivers of their adoption and their impact on the safety of food. Thus, this study examined the hygiene and handling practices of adult vegetable traders in southwest Nigeria by analyzing microbial contamination in vegetable samples and survey data from 166 traders collected over multiple seasons. Our findings show that just half of the traders routinely changed the washing water (every four hours), putting consumers at risk of severe *E. coli* infection. Almost no traders have received formal training on food safety. High toilet-use fees and a limited number of toilets are also significantly associated with practice gaps among traders. The study findings reveal the need for increased awareness about hygiene and food safety among food traders, e.g. through training programs. In addition, improving market infrastructure such as more toilets and hand washing stations and reducing the associated costs of using these services could facilitate better adoption and adherence to good hygiene practices which has a direct impact on food safety. Addressing food safety requires a multifaceted approach that includes education, infrastructure improvement, and policy interventions aimed at promoting and sustaining good food handling practices among food traders in Nigeria and similar contexts.

## Introduction

Food safety is a global challenge particularly critical in low- and middle-income countries (LMICs), where contaminated vegetables contribute significantly to foodborne illnesses and deaths. LMICs represent 75% of deaths from foodborne illness though they are only 41% of the global population (Havelaar et al., 2015; Grace, 2015a, 2015b; Kirk et al., 2015; Grace, 2023). In Africa, the per-capita burden of foodborne disease is about 27 times that of Europe or North America, but the health system has limited capacity for diagnosis and treatment (Havelaar et al., 2015; Morhason-Bello et al., 2013; Okoruwa & Onuigbo-Chatta, 2021). Traditional food markets are shared spaces (often informally arranged and managed by local authorities, traders themselves and/or community representatives) where people come together to trade food, goods and services. They are an important source of affordable food and livelihood to many in LMICs (Avijit Banik et al., 2020; FAO, 2007; Haque et al., 2024). However, traditional markets face heightened food safety challenges such as unreliable water supply and insufficient storage infrastructure, and a large share of food (raw and ready-to-eat) sold in many African food markets are contaminated at the point of purchase (Jaffee et al., 2018; Paudyal et al., 2017). This poses a significant threat to the public health of citizens. Despite this recognition, few studies have examined the drivers of good hygiene (e.g., washing hands after using the toilet) and food handling practices (e.g., sorting vegetables and routinely changing the water used to wash vegetables) among food traders and their impact on the safety profile of food in traditional markets. Thus, this study uses evidence from Nigeria, Africa’s most populous country to explore the drivers of good hygiene and food handling practices in traditional markets and their impact on the safety profile of fresh vegetables.

Nigeria is a transitioning LMIC where foodborne diseases cause approximately 173 million cases of diarrhoea, 33,000 deaths, and high mortality and morbidity costs exceeding USD 6 billion annually (Jaffee et al., 2018; Grace et al., 2018; Grace, 2015a, 2015b; Grace, 2016). Traditional food markets remain the main source of food for consumers across Nigeria (as in other African countries) and many low-income consumers depend almost exclusively on traditional markets for nutrient-rich foods like fresh vegetables (Adeosun et al., 2022; Fanzo et al., 2017). The presence of food safety challenges in Nigerian food markets (also common in other low-income countries) has been clearly documented (Nordhagen et al., 2022; Nordhagen et al., 2023; Okoruwa & Onuigbo-Chatta, 2021; Kuboka et al., 2024). There is also clear evidence of poor handling practices such as vegetable exposure to dust (by leaving uncovered) and washing and/or soaking vegetables in dirty water for extended period (Faremi et al., 2018; Makinde et al., 2020; Uchendu, 2018) in these markets. Whether open-air or enclosed, many traditional markets in many LMICs lack proper infrastructure, access to clean water, sanitary conditions, and adequate storage facilities, posing significant risks for the growth and spread of foodborne hazards (DeWaal et al., 2022; Mensah et al., 2002). Evidence indicates that a substantial portion of the food available in traditional markets is contaminated (Grace, 2015b) and consumer food preparation at home is often not effective in reducing the risk of foodborne illnesses (GAIN, 2020). Despite these findings, no studies the authors are aware of have brought together the extent and drivers of good hygiene and food handling practice adoption among fresh vegetable traders in Nigerian markets and its link to the presence of microbial contaminants in samples from the same traders. With information about what drives the adoption of good hygiene and handling practices and how these practices are linked to the food safety profile of the same handlers, we can link trader behavior and practices to food contamination and garner insights on potential mechanisms to encourage the adoption of important practices to increase the safety profile of food in traditional food markets. This study also contributes to a thin but growing body of evidence on the important role of actors in the midstream and downstream of food supply chains (such as wholesalers, retailers, food processors and transporters) promoting access to affordable, safe and nutritious foods (Reardon et al., 2021).

Fresh fruits and vegetables play an important role in a balanced diet. Public health emphasizes the importance of ensuring the safety of such foods given their potential impact on health worldwide regardless of age, race, gender, or income level (Gizaw, 2019). The popularity of fresh and ready-to-eat (RTE) fruit and vegetable consumption is growing due to their ease of preparation, nutritional value (e.g. in salads and smoothies), practicality, and appealing taste (Arslan & Ertürk, 2021; Xu et al., 2015). Fresh vegetables eaten as salads are rich in dietary fiber content, phytochemicals and antioxidants which will contribute to human health and well-being (Finger et al., 2023; Jideani et al., 2021). These foods, requiring no further heating or cooking, can be an important source of key nutrients (particularly minerals and vitamins). However, they are also potentially contaminated with pathogens (e.g. bacteria such as *Bacillus*, *Salmonella*, *Staphylococci*, *Escherichia coli (E. coli)* and *Listeria monocytogenes*) due to earlier mentioned factors such as poor hygiene and environmental factors in food markets. There is ample evidence regarding food contamination in traditional markets in LMICs. Majority focus on characterizing the presence of contaminants from food samples tested in laboratories (Imafidor et al., 2018; Eni et al., 2010; Ofor et al., 2009; Ehimemen et al., 2019; Erinle & Ajayi, 2022; Zwe & Yuk, 2017; Barreira et al., 2024; Tamiru et al., 2024). Other surveys or studies assessed traders’ knowledge of food safety and handling practices (Adane et al., 2018; Aljasir, 2023; Gemeda et al., 2023; Hamed & Mohammed, 2020) and/or their food handling practices (Akoachere et al., 2018; Aljasir, 2023; Mensah et al., 2002; Nizame et al., 2019; Solomon et al., 2018). To our knowledge, no studies have linked the practices of food vendors in traditional markets to contaminants present in the food being sold by those same vendors. Thus, this study contributes to filling this gap by explicitly exploring the extent and drivers of the adoption of good hygiene and handling practices (by vegetable traders) and its impact on the presence of microbial contaminants, *E. coli*, *Salmonella spp*. and *Bacillus spp.* in fresh vegetables in food markets. These pathogens (*E. coli*, *Salmonella spp*. and *Bacillus spp.*) are among the most prevalent foodborne pathogens which are of public health concerns and have been known to cause foodborne diseases such as diarrhoea, typhoid fever, dysentery and cholera, gardiasis, strongyloidiasis, and taeniasis etc. (Orpin et al., 2020). Pathogens such as *E. coli* are an important bacterial indicator of water quality and a major public health concern related to water and food safety (Devane et al., 2020). Good hygiene is captured by handwashing with soap and water (after bathroom use) while good handling practices include proper vegetable washing and the method of product display, which have been identified as significant sources of vegetable contamination (Ayensu, 2020).

This study adopts a multidisciplinary approach that leverages on research tools from the fields of applied economics and food science to identify contaminants in fresh vegetables and to understand their drivers. We use survey data collected from 166 food traders (visited multiple times to capture variation in temperature and water access across seasons) to understand the nature and drivers of hygiene and food handling practices among vegetable traders. We supplement this with a laboratory analysis of vegetable samples collected from each trader during each visit to test for microbial contaminants. We use a regression analysis that accounts for the fact that we have data from the same traders over multiple periods to identify the drivers of good hygiene and food handling practices as well as to test for statistically significant correlations between traders’ hygiene and handling practices and the presence of microbial contaminants in their vegetables.

This article is structured as follows: Sect. 2 describes the materials and methods used for this study while Sect. 3 presents our data and results. Section 4 is a discussion of our findings and Sect. 5 concludes with some recommendations and policy implications.

## Materials and methods

### Study area

This study was conducted in nine markets from the three senatorial districts (sub counties) in Ogun State, Southwest Nigeria. Ogun State is a Nigerian state with a high population density, especially in urban and peri-urban areas with over 50 million people and well known for its diverse agricultural activities including vegetable cultivation (Adekunle, 2012; Adeleye et al., 2020). We selected urban Ogun State because of its representation of typical food markets in Nigeria (compared to the large commercial capital (Lagos) or the administrative capital (Abuja), where extensive vegetable cultivation and consumption occurs. While this study is not nationally representative, the study sample reflects the conditions in many traditional markets in urban areas of southwestern Nigeria and also quite similar to food markets in urban Nigeria more broadly.

### Sample and Survey data collection

We administered a questionnaire to a sample of ~ 170 vegetable traders (selected using a random sampling method from a list of traders in the selected markets) to capture their demographic characteristics as well as their hygiene and food handling practices. We visited each trader three times; in March (end of the dry season), May (middle of the rainy season) and October (end of the rainy season) in the year 2023. During each visit, we collected vegetable samples from the traders of fresh tomatoes, carrots and cucumbers. These fresh vegetables are the most consumed vegetables due to their affordability and availability across all seasons (Raaijmakers et al., 2018). The schedule for data collection (March, May and October) was chosen to capture important seasonal variations in microbial contamination due to changes in temperature and water availability. In addition to collecting demographic information about the traders (e.g. age, gender, and level of education), we also collected information about their handling practices for their vegetables and about general hygiene (washing of hands after using the toilet) using a paper questionnaire. Recognizing the bias often associated with sensitive questions such as hygiene practices, enumerators were trained on strategies used to ask such questions via a conversation that was non-judgmental. Enumerators also supplemented information from respondents with their observation of trader practices at the markets. The questionnaire included multiple choice and open-ended questions adapted from standard validated questions used in Nigeria and was collected by enumerators that were trained on the instrument and engaged in a pilot test of the questionnaires in markets not included in the study.

To develop the sampling frame, a listing of markets from each of the 3 senatorial districts was done. Then three markets were selected randomly from each senatorial districts to capture variation across different sub counties among major markets with large sale volumes and serving large populations. Anecdotal evidence indicated that there are typically between 6 and 25 traders of our study commodities (tomatoes, carrots and cucumbers) in retail markets in urban Ogun state. Thus, we used the Yamane formula to calculate the sample size needed to attain an 8% margin of error.^1^ We assumed that the number of retailers of our three study products in markets in Urban Ogun state is about 5000. This indicated that we needed at least 151 observations to be able to test for statistically significant differences in decisions about handling practices. Traders in each market were listed and a sample of 175 selected randomly. During the first round of data collection, 174 traders were successfully interviewed. For the second and third rounds of data collection, 172 (98%) and 163 (93%) traders were re-interviewed. We confirmed that the main study findings are robust to the loss of 2 and 13 traders in rounds 2 and 3 respectively.^2^ We purchased samples of vegetables from every trader in our sample during each visit (giving us 498 samples from 166 traders visited in at least two rounds) and transported the vegetables in cold storage with a temperature of 4–6 ℃ to the laboratory (the Nigerian Institute of Medical Research (NIMR), Yaba, Lagos State) where they were analyzed for microbial studies. Each trader was selected for a particular product and two samples, each of carrot and tomatoes and one sample of cucumber, were collected from the different vegetable traders. This study (with **MSU Study ID:** STUDY00008064) was reviewed and approved by the Institutional Review Board of the lead partner University (Michigan State University) with concurrence from the partner institution (Federal University of Agriculture, Abeokuta). All interviews were conducted in accordance with Nigerian legislation and institutional requirements and all participants provided their informed consent to participate in this study after being given a description of the study.

### Microbial isolation and identification

For the microbial analysis, all glass wares used were washed thoroughly, air-dried and sterilized at 160 $^\circ{\rm C}$ for 1 h. The media used were sterilized in an autoclave at 121 $^\circ{\rm C}$ for 15 min. The bench working areas were swabbed with alcohol before any microbiological analysis to avoid contamination. One gram of sample was introduced into 9 ml of sterile peptone water in a pre-labelled sterile tube. The tube was incubated aerobically at 37 ℃ for 24 h. About 10 µL of inoculum was streaked onto a MacConkey agar (MAC) plate for *E. coli*, Salmonella shigella (SS) agar plate for *Salmonella spp*., and a Listeria agar plate for *Bacillus spp*. isolation and incubated aerobically at 37 °C for 24 h (Cheesbrough, 1985). The isolates were further sub-cultured. Suspected *E. coli* isolates from fresh MacConkey plate were sub-cultured by streaking onto MacConkey agar (MAC) plate, suspected *Salmonella* isolates from fresh Salmonella shigella (SS) agar plate were sub-cultured by streaking onto Chromogenic Salmonella agar and suspected *Bacillus* isolates from fresh Listeria agar plate were sub-cultured by streaking onto Chromogenic *Bacillus* agar. All plates were incubated aerobically at 37 °C for 24 h for presumptive confirmation as *E. coli*, *Salmonella spp.* and *Bacillus spp.* (Bergey & Holt, 1994).

### Empirical Analysis using multiple regression analysis

Descriptive statistics (means, and standard deviations frequency, percentage and cumulative frequency) were calculated for each variable and used to describe the study sample of traders, their hygiene and food handling practices and the prevalence of contaminants in the traders’ samples collected each period. This was followed by a statistical analysis of the drivers of trader adoption of good hygiene and handling practices as well as the correlation between the practices and presence of microbial contaminants.

Equation (1) represents the empirical model used for statistical analysis to explore the determinants of trader adoption of good hygiene and handling practices (what we refer to as step 1). The equation also determines the correlation (i.e. any statistical association) between these practices and the presence of microbial contaminants in step 2.

1 $${Y}_{it}={ \propto }_{i }+{{\varvec{\beta}}}_{1}{{\varvec{X}}}_{it}+{\mu }_{t}+{\varepsilon }_{it}$$

In Eq. 1, ${Y}_{it}$ is our outcome variable of interest which in step 1 is whether trader *i* visited in month *t* was using a good hygiene or handling practice or not. ${{\varvec{X}}}_{it}$ is a vector of trader and market level characteristics that could explain their adoption of the different practices, ${\propto }_{i}$ are time-invariant trader-specific effects, ${\mu }_{t}$ are month fixed effects (to capture variation in the adoption of these practices across seasons) and ${\varepsilon }_{it}$ is the idiosyncratic error term. ${{\varvec{\beta}}}_{1}$**,** (our primary focus in this analysis) is a vector parameter to be estimated to explain how the trader and market characteristics explain the observed variation in the adoption of good hygiene and food handling practices. For step 1, we estimate Eq. 1 using a pooled probit model given the binary nature of our adoption variables (taking a value of one if a trader reported using a particular practice and zero otherwise). The pooled probit is appropriate here to account for the fact that we have information from the same trader over multiple periods but there is limited variation within traders in their use of the different handling or hygiene over the three survey rounds and in their demographic characteristics.

For step 1, the five good hygiene/handling practices that we consider are (1) routine water changes every 4 h, (2) sorting vegetables after purchase to remove those that are of lower quality or bad (3) using a plastic crate to move or store their products (4) if the trader separates their vegetables and (5) if traders adopt good hygiene practices measured as whether they wash their hands with soap and water after using the bathroom (Rayza et al., 2016; Akoachere et al., 2018; Solomon et al., 2018; Nizame et al., 2019; Hamed & Mohammed, 2020; Aljasir, 2023; Boakye et al., 2023; Magqupu et al., 2024). The selection of these practices was drawn from a review of the literature on food safety. The explanatory variables considered include trader demographic variables (e.g. gender, age, education) a proxy for the scale of the trader (i.e. the volume of the traders last transaction), what product the trader sold (i.e. tomatoes, cucumber or carrots) and market level characteristics such as the location of the market (senatorial district), the number of toilets, the cost for water and or toilet use in the markets.

In Step 2, ${Y}_{it}$ (in Eq. 1) is our outcome variable of interest (i.e. whether a sample of vegetables purchased from trader *i* in month *t* had a particular contaminant or not) and we explore three food microbial contaminants: *E. coli*, *Salmonella spp*. or *Bacillus spp*. Here, ${{\varvec{X}}}_{it}$ is a vector of trader and market level characteristics as for step 1 as well as trader hygiene practices (e.g. washing hands after using the toilet) and handling practices (e.g., sorting and routinely changing wash water) that could explain the variation in the presence of contaminants in trader vegetable samples. As in Step 1, ${\propto }_{i}$ are time-invariant trader-specific effects, ${\mu }_{t}$ are month fixed effects and ${\varepsilon }_{it}$ is the idiosyncratic error term. Here, ${{\varvec{\beta}}}_{1}$ is a vector of parameters to be estimated associated with trader and market characteristics explaining the presence of microbial contaminants. For Step 2, we estimated a random effects probit model which allows us to account for the fact that we have information from samples collected from the same trader over multiple periods with significant variation in the presence of contaminants in samples from the same trader at different times and limited variation in trader and market characteristics. We recognize that a random effects probit estimation of the impact of handling practices on microbial contaminants does not account for unobservable factors that could be correlated with both traders’ adoption of good practices and their likelihood of preventing contamination of the vegetables they sell. Because most trader characteristics and hygiene/handling practices do not change over time, we are unable to fully exploit within trader variation over time and thus conduct a between trader analysis. We do not interpret our findings as causal but rather as important and informative correlations (or statistical associations) between trader handling/ hygiene practices and the presence of microbial contaminants, conditional on a rich set of explanatory variables (at trader and market level) that could jointly determine the trader practices and product contamination. In all estimations, we cluster our standard errors at the trader level to account for likely serial correlation of trader behavior over survey rounds and confirm that our key conclusions in both steps are robust to the use of the pooled estimator or the random effects probit estimator.^3^

## Results

### Descriptive statistics of vegetable traders

Table 1 presents the descriptive statistics of the study sample. Sixty percent of the vegetable traders were female with an average age of 39 years, about 16 years of experience in selling vegetables and 16% of traders having no form of education. The sample was similarly distributed across the three senatorial districts with a slightly higher share engaged in trading tomatoes (45%) compared to cucumbers (30%) and carrots (25%). Very few traders (< 1%) had ever attended a training related to food safety and all traders were in a market with a toilet available within a 5-min walk costing on average about ₦ 65 ($0.10 in 2023 at N700 = $1).

**Table 1:** Descriptive statistics of vegetable traders in the study sample (*n* = 166)

Variable	Mean
Male (Yes = 1, No = 0)	0.40
Years of selling vegetables	15.76[11.14]
Ever attended food safety training (1/0)	0.004
Cost of using a toilet in the market in naira (dollars)	64.36 ($0.10) [46.72]
Cost of water in the market per liter in naira (dollars)	91.05 ($0.13) [28.82]
Number of toilets in the market	2.89[2.20]
Ogun Central	0.36
Ogun East	0.34
Ogun West	0.30
Tomatoes	0.44
Carrots	0.22
Cucumbers	0.32
Age in years	39.34[13.04]
Quantity of vegetables purchased in last transaction (kg)	43.75[39.33]
Trader has less than primary education (1/0)	0.16

Standard deviations are included in the square brackets []

### Share of vegetable samples with microbial contamination

**Fig. 1:**
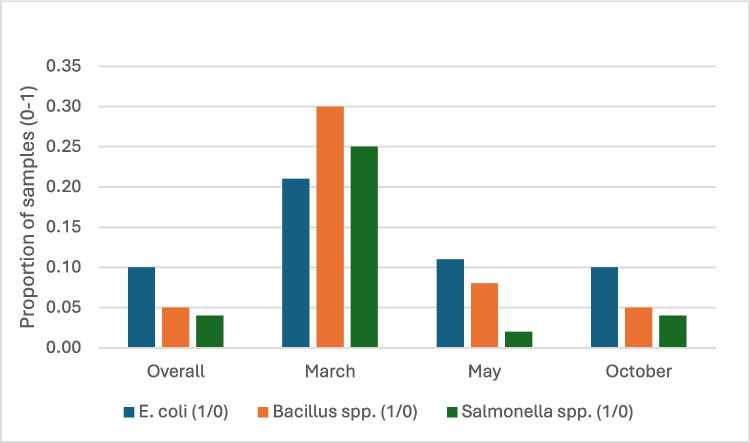
Share of fresh vegetable samples with different microbial contaminants by season (*n* = 166)

**Fig. 2:**
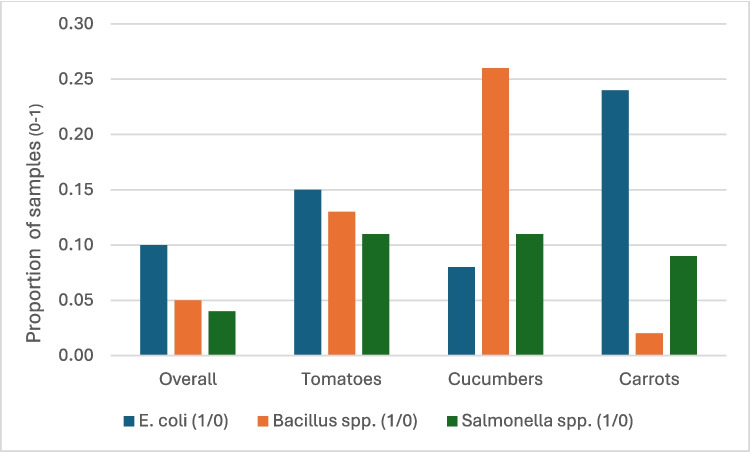
Share of fresh vegetable samples with different microbial contaminants by vegetable (*n* = 166)

Figures 1 and 2 present the share of samples purchased from the traders that tested positive for the presence of the three study microbial contaminants (by season and vegetable type) and highlights several key points. First, the share of samples with the identified pathogens were significantly higher at the end of the dry season (round 1) compared to the middle and end of the rainy season (rounds 2 and 3). The share of vegetable samples with *E. coli* in March was about double the share from samples collected in May and October. The share of samples with *Bacillus spp.* and *Salmonella spp.* reduced by a third from 30 and 25% to 8% and 2% respectively between March and May. By October, the proportion of samples with contaminants remained significantly lower compared to March and comparable to the level observed in May. *E. coli* contamination was notably more common in carrot samples (24%) than in tomatoes (15%) and cucumbers (8%). Conversely, *Bacillus spp*. was more prevalent in cucumber samples (26%) compared to tomatoes (13%) and carrots (2%). The incidence of Salmonella spp. was consistently lower than that of *E. coli* and *Bacillus spp*., averaging 10% across all three vegetables.

### Adoption rates of good hygienic and handling practices among traders

Table 2 presents the adoption rates of good hygiene and handling practices among traders segmented by product type—tomatoes, carrots and cucumbers. The majority of the vegetable traders (95%) sorted their vegetables after purchase (to remove the bruised and damaged vegetables) and sold their vegetables on a raised platform (83%) vs. placing it on a sheet/sack on the floor. Eighty percent of the traders in our sample separated their vegetables while on display. Most traders (85%) reported washing their hands with soap and water after using the toilet, and half of them (50%) changed the washing water within 4 h. However, only 10% used plastic crates for storage or transportation.

**Table 2:** Adoption rates of good hygiene and vegetable handline practices among traders (*n* = 166). All variables are expressed as proportions

	Overall	Tomatoes	Cucumbers	Carrots
Changes water ≤ 4 h	0.52	0.75	0.35	0.34
Uses a plastic crate to store/ move product	0.12	0.24	0.01	0.04
Has good hygiene practices	0.87	0.98	0.85	0.76
Separates vegetables for display	0.80	0.89	0.74	0.76
Vegetables are sold on a raised platform	0.83	0.77	0.94	0.84
Sorts vegetables after purchase	0.95	1.00	0.91	0.90

We found adoption rates for most practices to be higher among tomato traders. For example, while 98% of tomato traders reported good hygiene practices, this was lower among cucumber (85%) and carrot (76%) traders. Similarly, while 75% of tomato traders noted that they changed their washing water within 4 h of first use, this was done by only 35% of cucumber and carrot traders. The adoption rate of plastic crates was 25% among tomato traders, compared with less than 5% for carrot and cucumber traders.

### Determinants of the adoption of good hygienic practices

Table 3 presents the pooled probit results on the factors associated with the adoption of good handling and hygienic practices among vegetable traders. Five key points emerged. First, it was observed that male and older traders were more likely to have reported changing their washing water appropriately. Second, a non-linear relationship between the quantity of vegetables purchased in the traders’ last transaction (i.e. their last purchase of vegetables from their supplier for the batch that the sample taken was purchased) and the likelihood of changing washing water routinely was also observed. Traders who purchased larger quantities were more likely to change their washing water correctly, but this effect reduces as the quantity purchased increases.

**Table 3:** Determinants of the adoption of good hygiene practices

	(1)	(2)	(3)	(4)	(5)	(6)
	Changes water ≤ 4 hours	Uses a plastic crate to store/ move product	Has good hygiene practices	Separates vegetables for display	Vegetables are sold on a raised platform	Sorts vegetables after purchase
Male trader (1/0)	0.176**	−0.181***	−0.125***	−0.021	−0.037	0.081
Age of the trader (years)	0.009***	−0.001	0.002	0.001	0.002	−0.003
Years of selling vegetables	0.001	0.001	0.001	−0.003	0.004	0.006
Vegetable quantity purchased in the **last** transaction from which sample was taken (kg)	0.009***	0.000	−0.011***	−0.006***	−0.005***	−0.005**
Squared quantity of vegetables purchased in last transaction from which sample was taken (kg)	−0.000***	−0.000	0.000***	0.000***	0.000***	0.000
Trader has less than primary education (1/0)	−0.051	0.030	−0.045	−0.054	0.032	−0.042
Cost of water in the market (naira)	−0.001	-	-	-	-	-
Ogun Central (1/0)	0.258***	−0.162***	−0.002	0.153**	−0.113	−0.003
Ogun East (1/0)	0.104	−0.088***	−0.037	0.173**	−0.199***	0.129*
Ogun West (base)	-	-	-	-	-	-
Trader sells tomatoes (1/0)	0.205***	0.124***	0.084*	0.182**	−0.042	
Trader sells cucumbers (1/0)	−0.075	−0.085	0.039	−0.012	0.153**	0.057
Trader sells carrots (base)	-	-	-	-	-	-
Number of toilets in the market			0.115***			
Cost of using a toilet in the market (naira)			−0.032***			
Number of observations	496	496	496	496	496	496

Third, female traders and tomato traders were more likely to report good hygiene practices while traders dealing in larger quantities were less likely to have reported good hygiene practices, all things being equal. Traders in a market with more toilets and lower toilet-use-fees are more likely to have reported good hygiene practices. Fourth, traders dealing with larger volumes were less likely to sort and market location matters. Traders in markets in Ogun East were also more likely to report sorting compared to those in Ogun West and Ogun Central. Similar to vegetable sorting, traders with larger volumes purchased in their last transaction were less likely to separate their vegetables for display or use a raised platform. Fifth, the adoption of plastic crates was higher among female and tomato traders consistent with the descriptive statistics. The likelihood of plastic crate being used was significantly lower among traders in Ogun East and Ogun Central compared to those in Ogun West.

### The relationship between good handling practices and microbial contamination in vegetable samples

Table 4 presents the results of the random effect probit estimation on the correlation between good hygienic and handling practices and the presence of microbial contaminants in vegetables sold by traders. A key finding is that changing washing water properly was associated with a significantly lower probability of consumer exposure to *E. coli*. Samples from traders who reported changing water properly (within 4 h) were associated with about 10 percentage points lower probability of testing positive for *E. coli* (column 1). There was limited evidence of handling practices being associated with contaminants for *Salmonella spp*. and *Bacillus spp*. No handling practices were associated with *Salmonella spp*. being present in a sample and the only handling practice found to be significantly associated with the probability of finding *Bacillus spp*. in a sample was the use of a plastic crate to store or transport the vegetables. Using a plastic crate is negatively associated with the probability of finding *Bacillus spp*. in a sample and this is statistically significant at 10%.

**Table 4:** The relationship between good handling practices and microbial contamination in vegetable samples (Average Partial Effects-APE)

Explanatory variables	*E. coli*	*Salmonella spp.*	*Bacillus spp.*
Male trader	0.084*	0.068	0.020
Age of the trader	0.002	0.001	0.003**
Years of selling vegetables	−0.002	0.001	−0.003*
Sample gotten in May	−0.104***	−0.223***	−0.216***
Sample gotten in October	−0.114***	−0.199***	−0.231***
Sample gotten in March (base)	-	-	-
Trader changes water within 4 h of first use	−0.101**	0.030	0.026
Trader sorts their vegetables after purchase	0.082	0.045	0.032
Vegetable quantity purchased in last transaction (kg)	0.001	−0.001	0.001
Squared vegetable quantity purchased in last transaction (kg)	−0.000	−0.000	−0.000
Trader has less than primary education	0.024	0.001	0.007
Trader uses plastic crates to store and transport product	−0.006	0.002	−0.114*
Trader demonstrated good hygiene practices	−0.101*	−0.008	−0.031
Trader separates vegetables on display	0.088*	0.021	0.051
Trader uses a raised platform	−0.067	−0.013	−0.024
Ogun Central Senatorial District	0.086*	0.020	−0.002
Ogun East Senatorial District	0.038	0.078*	−0.101*
Ogun West Senatorial District (base)	-	-	-
Tomato sample	−0.018	0.017	0.213***
Cucumber sample	−0.138***	0.029	0.309***
Carrot sample (base)	-	-	-
Number of observations	496	496	496

Seasonal variations, trader characteristics, and vegetable types influence the probability of microbial contamination in vegetable samples. The study reported a higher contamination rate by *E. coli*, *Salmonella spp*. and *Bacillus spp*. in vegetable samples at the end of the dry season compared to the rainy season. Samples collected from male traders were more likely to have tested positive for the presence of *E. coli*, all things being equal, and this result is statistically significant at 10%. Additionally, samples from older traders and those with less trading experience were more likely to be contaminated with *Bacillus spp*. Samples from cucumber traders were about 14% points less likely to have tested positive for *E. coli* compared to samples from carrot traders. Tomato samples were more likely to have tested positive for *Bacillus spp*. compared to carrots.

## Discussion

This section aims to provide explanations about the observed correlations between trader practices and the presence of microbial contaminants in their vegetable samples. We observed double the share of samples with microbial contaminants in the dry season compared to the rainy season. This is likely due in part to higher temperatures promoting microbial growth (Ruiz et al., 1987). Other potential contributing factors include limited water sources during the dry season, low water level of wells, and raw sewage discharge (DeJager et al., 2022). Higher microbial contaminant presence during the dry season is consistent with findings from other studies noting significantly higher coliform organisms in the dry season than in the wet season (Agbogu et al., 2006; Okafo et al., 2003). A study by Muniz et al. (2020) also reported higher rates of *E. coli* during the dry season compared to the rainy season. *E. coli* contamination was notably higher, reflecting poor water quality and inadequate washing, compared to the other contaminants. It is important to note that washing vegetables with water contaminated with *E. coli* can also contaminate the vegetables. Thus *E. coli* contamination could be driven by multiple factors in traditional markets if washing is not done properly and/or the quality of water is poor. Our findings are consistent with Prayoga et al. (2021) who found high levels of E.coli in markets in two different sites; Huong Chu and Phu Mau in Hue in Vietnam. The high presence of *E. coli* detected in our carrot samples may be because of contamination from animal or human waste residues in the soil (Lynch et al., 2009) and evidence of no or poor washing. This is a particular concern in the Nigerian context where fresh carrots are often consumed directly after purchase from traders, but most markets have poor infrastructure and lack access to good water supply.

Our results indicated significant heterogeneity in the adoption rates of good hygiene and handling practices across the study sample. The adoption of good hygiene practices and all good handling practices except selling their products on a raised platform was much higher among tomato traders compared to cucumbers and traders. While tomatoes are often cooked further in Nigerian cuisine, cucumber and carrots are often consumed fresh and without further processing, with carrots sometimes consumed immediately upon purchase. This indicates the need for particular attention to be paid to encourage the adoption of good hygiene and handing practices by traders of carrots and cucumbers.

The use of plastic crates was low, potentially due to cost and familiarity with traditional baskets, though the crates were linked to lower contamination by *Bacillus spp*., a soil bacterium (Aghadi et al., 2020). Our findings revealed that the practices of properly washing vegetables with water was more common among older traders, male traders and traders who purchased larger quantities of vegetables. Our finding that these practices reduce consumer exposure to food borne pathogens is consistent with several other studies that have reported that properly washing of vegetables with water is key for preventing contamination and can significantly reduce fecal contamination (Moris & Brady, 2005; Amoah et al., 2005). We found that female traders and tomato traders were more likely to have reported good hygiene practices which ultimately reduce consumer exposure to microbial contaminants. Gender differences in behavior is consistent with Mohammed et al. (2020) who also found that female traders tend to practice better hygienic practices than their male counterparts attributed to their experience in managing homes.

We found that traders in markets with more toilets and lower toilet-use-fees were more likely to have reported good hygiene practices. Since markets in the study area typically provide water and soap for handwashing (as part of the toilet-use-fee), these findings suggest that more toilets near the vending area would increase access for traders and reduce the effort needed to reach the toilets and the associated services. However, higher costs of using the toilet might encourage traders to use other avenues for defecation such as plastic bags or the bush which are less likely to come with water and soap for handwashing.

The significant correlation between the use of plastic crates and lower levels of *Bacillus spp*. is consistent with the fact that *Bacillus spp*. is a genus designated as a group of soil inhabitants and was likely prevented with the use of plastic crates. The limited impact of handling practices on the presence of *Salmonella spp.* and *Bacillus spp*. is likely due to their generally low presence (5%) in our samples. We find that samples taken from traders who separated their products (rather than mixed them together) were more likely to have *E. coli*. This might not be surprising due to increased handling and exposure of separated products to contaminants, including greater consumer contact, improper washing practices, lack of protective packaging, and less hygienic storage conditions. Observation during market visits and anecdotal evidence from interacting with traders indicated that sometimes when products are separated and unpackaged, they entice more consumers to touch them during the product search and negotiation process than when products are all mixed together.

The likelihood of microbial contaminants varies with trader characteristics and vegetable type. Vegetable samples from older traders and those with limited trading experience were more likely to be contaminated with *Bacillus spp*. This might reflect older traders’ reluctance to use new practices such as plastic crates or modern packaging that are contrary to their traditional practices and is consistent with the literature that has shown that older people are often less willing to adopt new practices (Özsungur, 2019). In contrast, longer trading experience may help traders implement effective strategies to keep their products clean and safe over time. Tomato samples were more likely to have tested positive for *Bacillus spp*. compared to carrots. The higher contamination of tomato samples by *Bacillus spp*. may be because *Bacillus spp*. is predominantly a soil bacterium, and these findings might reflect the inadequacy of current washing practices to completely remove the soil residues on tomatoes. While tomatoes are increasingly being consumed raw in Nigeria in salads and smoothies, this finding might reflect that less attention in washing is given to tomatoes because they are expected to be further processed at homes (to make stews and sauces) compared to cucumbers and carrots that are more widely considered ready for immediate consumption. In addition, tomatoes are often grown in conditions that may involve the use of *Bacillus thuringiensis*-based insecticides which can leave residues on the fruit's surface and contribute to increased contamination levels. A study by Frederiksen et al. (2006) identified the presence of *Bacillus spp*., including *B. thuringiensis*, on fresh produce such as tomatoes, cucumbers, and peppers, likely because of these insecticide applications.

Overall, this article found a significant correlation between trader handling and hygiene practices and contaminant levels in vegetables sold in Nigerian food markets. However, the adoption of several recommended practices remains low. Efforts to inform and encourage traders about the importance of properly washing their vegetables and good hygiene practices could significantly reduce consumer exposure to microbial contaminants in fresh vegetables and other RTE food that may only be lightly cooked or consumed raw to preserve their nutrient content. A key finding is that while the majority of traders wash their vegetables, it is crucial to change the wash water frequently– washing vegetables is not enough, but it is important that the water used to wash the vegetables is changed frequently, currently only done by 50% of the study sample.

## Conclusion and policy implications

This study explored the extent and drivers of good hygiene and food handling practices among vegetable traders in traditional food markets in Ogun State, Southwest Nigeria. Using descriptive statistics and a multivariate regression analysis, we found that microbial contaminant levels in study samples of vegetables were much higher in the dry season compared to the rainy season. While washing vegetables was done by over 95% of traders, only half of the traders used the recommended practice of changing washing water regularly (every 4 h). This recommended practice was observed to significantly reduce the contamination rate of *E. coli* in vegetable samples. The majority of traders sold their fresh vegetables in open spaces and displayed unpackaged on tables or in wheelbarrows exposing them to contaminants by poor hygiene and handling practices of both vendors and customers. As expected, good hygiene practice such as washing hands with soap and water after toilet use, was found to be negatively associated with the presence of *E. coli.*

Though good hygiene and food handling practices can reduce food contamination, almost no traders in our sample had ever received any training on food safety related issues. Efforts to increase awareness about hygiene as well as incentives and/or enforced regulations around hygiene and food handling practices are needed. This study concludes that while improving the safety of vegetables might be complex because of the numerous potential sources of contaminants, there are some relatively simple strategies that can significantly improve the food safety profile of foods in traditional African markets. First, more trainings/awareness campaigns around good hygiene and food handling practices such as appropriate washing are needed. Second, it is important to consider the value and types of simple packaging of fresh products such as vegetables, even without fancy labels to prevent them being touched by customers or traders.

For the effective adoption of good hygiene and handling practices, market leaders, government and donors should leverage on trader characteristics found to be associated with the adoption of good practices (e.g. gender, age and scale) to design and target trainings and campaigns. For example, governments with limited resources may initially focus on designing and disseminating information on good hygiene and handling practices to traders particularly males and older traders currently less likely to be using/adopting the good practices. In addition, engagement with groups of food traders that tend to adopt good practices could inform on the development of strategies to encourage their more widespread adoption. Finally, governments, market leaders and donors can support broader adoption of good hygiene and handling practices via efforts to improve market infrastructure (such as provision of more toilets and hand stations and good water supply) and by reducing the cost of toilet use in food markets.

## Data Availability

Data for this study is available upon request.

## Footnotes

1 A margin of error between 4 and 8% is considered acceptable when using a 95% confidence level in most research studies with explanatory analysis such as is done in this study given budgetary considerations and the fact that traders were visited three times.

2 Restricting the analysis to rounds 1 and 2 maintains 98% of the initial sample while still allowing us to capture variation across seasons in the incidence of microbial contaminants as well as the correlation between trader practices and the presence of these contaminants. We also confirmed that our main findings are robust to consideration of the entire sample or only those traders retained across all study rounds. These results are available upon request. We present the results from the 166 traders with complete information for the key study variables in at least two rounds.

3 While our study findings are similar when using the random effects probit and logit models, the probit model is our preferred specification because of the ease of interpreting our results as average marginal effects (i.e. the effect of a unit change in a continuous explanatory variable (or its presence/adoption for binary variable) on the probability of each outcome variable being true. In moderate size datasets, the probit link often gives an improved fit over the logit link.
